# Implicit Solvent Models and Their Applications in Biophysics

**DOI:** 10.3390/biom15091218

**Published:** 2025-08-23

**Authors:** Yusuf Bugra Severoglu, Betul Yuksel, Cagatay Sucu, Nese Aral, Vladimir N. Uversky, Orkid Coskuner-Weber

**Affiliations:** 1 Molecular Biotechnology, Turkish-German University, Sahinkaya Caddesi No. 106, Beykoz 34820, Istanbul, Turkey; e200102055@stud.tau.edu.tr (Y.B.S.); e210102045@stud.tau.edu.tr (B.Y.); e180102030@stud.tau.edu.tr (C.S.); aral@tau.edu.tr (N.A.); 2 Department of Molecular Medicine and USF Health Byrd Alzheimer’s Research Institute, Morsani College of Medicine, University of South Florida, Tampa, FL 33612, USA

**Keywords:** implicit solvent models, biomolecular simulations, Poisson–Boltzmann equation, Generalized Born model, protein–ligand binding

## Abstract

Solvents represent the quiet majority in biomolecular systems, yet modeling their influence with both speed and ri:gor remains a central challenge. This study maps the state of the art in implicit solvent theory and practice, spanning classical continuum electrostatics (PB/GB; DelPhi, APBS), modern nonpolar and cavity/dispersion treatments, and quantum–continuum models (PCM, COSMO/COSMO-RS, SMx/SMD). We highlight where these methods excel and where they falter, namely, around ion specificity, heterogeneous interfaces, entropic effects, and parameter sensitivity. We then spotlight two fast-moving frontiers that raise both accuracy and throughput: machine learning-augmented approaches that serve as PB-accurate surrogates, learn solvent-averaged potentials for MD, or supply residual corrections to GB/PB baselines, and quantum-centric workflows that couple continuum solvation methods, such as IEF-PCM, to sampling on real quantum hardware, pointing toward realistic solution-phase electronic structures at emerging scales. Applications across protein–ligand binding, nucleic acids, and intrinsically disordered proteins illustrate how implicit models enable rapid hypothesis testing, large design sweeps, and long-time sampling. Our perspective argues for hybridization as a best practice, meaning continuum cores refined by improved physics, such as multipolar water, ML correctors with uncertainty quantification and active learning, and quantum–continuum modules for chemically demanding steps.

## 1. Introduction

Solvation phenomena profoundly influence the structure, dynamics, and function of biomolecules in aqueous and non-aqueous environments [1]. In biological systems, water and other solvents mediate essential processes such as protein folding, ligand binding, molecular recognition, and catalysis [2,3]. Accurately modeling solvation is important in computational biophysics and chemistry, with implications ranging from fundamental molecular science to practical drug discovery and (bio)material design [4]. Traditionally, explicit solvent models, where each solvent molecule is treated as a discrete particle, have been the gold standard for capturing solvation effects in molecular simulations. While explicit approaches provide detailed insights into solvent structure and dynamics, their high computational cost and the need for extensive sampling have motivated the development of alternative strategies [5]. Among these, implicit solvent models have emerged as crucial tools, offering a balance between computational efficiency and physical realism by replacing discrete solvent molecules with a continuum representation [6].

The conceptual foundations of implicit solvent models can be traced to early dielectric theories of solvation. The seminal work of Onsager and Debye in the early 20th century established the treatment of solvents as dielectric continua, enabling the estimation of solvation energies based on bulk properties such as dielectric constant and molecular polarizability [7]. These early models laid the groundwork for the development of more sophisticated theoretical frameworks [8]. With the advent of computational chemistry, the need for practical and generalizable solvation models led to the development of continuum electrostatic approaches [9]. The Poisson–Boltzmann (PB) equation provided a rigorous description of electrostatic interactions between solutes and a surrounding dielectric medium, representing spatial variations in dielectric properties and ionic strength [9,10]. Furthermore, the Generalized Born (GB) model introduced efficient pairwise approximations to the PB formalism, enabling the rapid estimation of electrostatic solvation energies for large biomolecular systems [9,11]. In parallel, advancements in quantum chemistry drove the creation of implicit solvation models such as the Polarizable Continuum Model (PCM) and the Conductor-like Screening Model (COSMO), which facilitated the inclusion of solvation effects in electronic structure calculations [12,13]. More recent models, such as the SMx family and SMD, integrate both electrostatic and non-electrostatic (cavitation, dispersion, and repulsion) contributions to provide highly accurate predictions of solvation free energies across a wide range of solvents and solutes [12,14,15].

At the center of implicit solvent models lies the partitioning of the solvation free energy into physically meaningful components [9]. Typically, this includes a polar (electrostatic) term, accounting for the interaction of the solute’s charge distribution with the dielectric environment, and a nonpolar term, describing contributions from cavity formation, solvent-accessible surface area, and van der Waals interactions [9,16,17,18]. These terms have been refined to incorporate additional physical effects, such as hydrogen bonding corrections and solvent-excluded volume [17]. Implicit solvent models are grounded in continuum theories, where the solute is embedded in a dielectric medium characterized by macroscopic properties. The electrostatic component is frequently computed by solving the Poisson–Boltzmann equation or its linearized form, or by employing the Generalized Born approximation. The nonpolar component is often related to the solvent-accessible surface area (ASA) or volume (SAV), with empirical parameters derived from experimental data or explicit solvent simulations [18,19,20,21]. Modern models further decompose the nonpolar term to distinguish between repulsive (cavity) and attractive (dispersion) interactions [18,22,23]. The primary advantage of implicit solvent models lies in their computational efficiency [9,18,24]. By removing the need to simulate thousands of explicit solvent molecules, these models enable the rapid exploration of biomolecular conformations, facilitate enhanced sampling, and make feasible the simulation of large or complex systems, which would be otherwise impossible. Thus, implicit solvent models have been widely adopted in biomolecular simulations, structure-based drug design, protein–protein and protein–ligand binding studies, and nucleic acid research [9]. However, the continuum approximation introduces inherent limitations. The absence of explicit solvent structure can lead to inaccuracies in capturing specific solvent-mediated interactions, such as water bridges, hydrogen bonds, and ion effects. Implicit models may also struggle to represent the entropic contributions of solvent molecules and the heterogeneous nature of biological environments [5,25]. Parameterization remains a challenge, as the accuracy of these models depends strongly on the choice of atomic radii, dielectric constants, and empirical coefficients [26]. Despite these challenges, ongoing refinement and the integration of machine learning (ML) techniques continue to enhance the reliability and predictive power of implicit solvent approaches [27].

The versatility of implicit solvent models has catalyzed their adoption in a wide array of biophysical applications [9]. In protein–ligand binding, implicit solvation methods are routinely employed to estimate binding free energies, rank inhibitor potency, and guide lead optimization [9]. For intrinsically disordered proteins (IDPs) whose lack of stable tertiary structure challenges traditional modeling, implicit solvents enable the efficient exploration of vast conformational landscapes [28,29,30] and facilitate comparison with experimental data such as FRET or SAXS [31,32]. In nucleic acid simulations, hybrid explicit and implicit solvent approaches have yielded accurate predictions of DNA and RNA structure and dynamics, with significant reductions in computational cost. Moreover, advances in quantum implicit solvent models allow for the incorporation of solvation effects into the ab initio calculations of reaction mechanisms, spectroscopic properties, and electronic structure predictions [12]. This has implications for the study of enzyme catalysis, photochemistry, and (bio)material design [12,33].

Here, we provide an overview of implicit solvent models, detailing their theoretical foundations, practical implementations, and applications in biophysics and computational chemistry. We describe classical and quantum formulations, benchmark studies, and recent developments in model parameterization and hybrid approaches. Special attention is given to recent ML-augmented implicit solvent models and their impact on accuracy and transferability. We also discuss current challenges and highlight future directions, including the integration of quantum computing and multi-scale modeling. By elucidating both the strengths and limitations of implicit solvent models, we aim to provide a critical resource for the selection and application of appropriate solvation models in diverse biophysical and (bio)chemical investigations.

## 2. Classical Implicit Solvent Models

### 2.1. The Beginning of an Era

The Onsager model was introduced by Lars Onsager [34] to refine Lorentz local field approximation in a system of dipoles [35]. The equation for Debye’s dielectric constant *ε* is given by

(1) $$\frac{\varepsilon -1}{\epsilon +2}=\frac{4\pi }{3}\sum_{i}N_{i}\left(\alpha_{i}+\frac{\mu_{i}^{2}}{3k_{B}T}\right),$$

where $N_{i}$, $\alpha_{i}$ and $\mu_{i}$ are the concentrations given by the number of molecules per unit volume (molecules/cc), the polarizability, and the permanent dielectric moment of the molecule of type i, respectively. The summation runs over all the different types of included molecules [34]. Debye’s equation failed in predicting pure polar liquids when compared with experiments. In Kirkwood’s work [36], it was observed that for dilute solutions of polar substances in nonpolar liquids, the dipole moments calculated using the theoretical formula closely matched those measured in the vapor phase, though there was a systematic trend for the moment (p) to decrease as the dielectric constant of the environment increases. However, for pure polar liquids, the calculated dipole moments are significantly smaller, and this discrepancy becomes more pronounced as the dielectric constant of the liquid rises.

Although its definition and content have changed over time [9,16,17,18,24,37], the solvation free energy, $∆G_{solv}$, plays a vital role in the thermodynamical, physical description of a solution [9,24]. In order to explain the free energy of solvation in a comprehensible way, these differences will be discussed in detail. Here, we first introduce the nonpolar free energy of solvation and then the other approaches that may or may not incorporate a nonpolar free energy component. Nonetheless, $∆G_{solv}$ is composed of two main components [5,17,37], polar and apolar, which are denoted by different appellations [5,16,37,38]. Electrostatic free energy of solvation, $∆G_{ele},$ is used to refer to electrostatic interactions between solute and solvent [9,16,24,37,38], whereas $∆G_{np}$ denotes nonpolar interactions:

(2) $$∆G_{solv}=∆G_{np}+∆G_{ele}$$

In parallel, $∆G_{solv}$ can be separated into three main components [9,24,39,40]. Firstly, $∆G_{cav}$ is used to define the excluded volume and the cavity of the solvent around the solute [9,24,40,41]; $∆G_{ele}$ is used to refer to electrostatic interactions between solute and solvent [9,24]; and finally $∆G_{vdW}$ describes the van der Waals interactions [9,24]:

(3) $$∆G_{solv}=∆G_{cav}+∆G_{ele}+∆G_{vdW}$$

Tan et al. stated that the nonpolar component of the solvation free energy $∆G_{np}$ consisted of two components with highly different interactions, namely, repulsive free energy and attractive free energy, and therefore these had to be modeled separately [18]. Gonçalves et al. introduced short-range free energy instead of crude solvation free energy of the van der Waals interactions $∆G_{vdW}$, which incorporates small-scale attractive and repulsive forces between solute and solvent molecules [24]. Before, it was thought that in transferring a molecule from the gas phase to the solvent, the cavity could be treated as spherical, as referred to in Equation (4) [42]:

(4) $$∆G_{solv}=4\pi r^{2}\sigma -\epsilon ,$$

where r is the cavity radius, σ is the solvent surface tension, and ϵ is the solute–solvent interaction energy [42]. However, as mentioned above, the solvation free energy has polar and nonpolar components [16,20], yet these components and later their sub-components are only relevant in further developments of implicit solvent models [9,16,17,18,20,43]. Next, accessible surface area (ASA) was defined [44] as the sum of the area around the atom of a molecule that solvent molecules can interact with without penetrating any other atoms of the molecule. For calculating ASA, different methods have been introduced [45,46,47]. However, ASA calculations required that buried atoms be considered as they could not contribute to the free energy of solvation [47,48]. The ASA for an atom i and its atomic solvation parameter $∆\sigma_{i}$ of the free energy of transfer is (aqueous solution) defined as follows [48,49]:

(5) $$∆G_{i}=∆\sigma_{i}ASA_{i}$$

where the sum of free energy transfer for a whole molecule of interest $∆G_{R}$, such as a residue of a protein, can be described as

(6) $$∆G_{R}=\sum_{i}∆\sigma_{i}ASA_{i}$$

However, we have to mention that polar interactions are omitted, and the calculation solely depends on ASA. Moreover, atomic solvation parameters vary for each atom subtype. Thus, ASA parametrizations were then conducted [9,48,49]. Later, Stil et al. was proposed that nonpolar solvation, the cavity, and van der Waals interactions can be defined as functions of ASA [50]:

(7) $$G_{cav}+G_{vdW}=\sum_{k}\sigma_{k}{ASA}_{k},$$

where $ASA_{k}$ is the total solvent-accessible surface area of atoms of type k and $\sigma_{k}$ is an empirical atomic solvation parameter. $∆G_{np}$ is a key component of $∆G_{solv}$; thus, nonpolar solvation free energy can be defined in terms of ASA [18]:

(8) $$∆G_{np}=\gamma ASA+c$$

where γ represents surface tension coefficient and nonpolar energy representation per unit surface area and c is a constant that defines nonpolar solvation energy. However, it has been addressed that, for small hydrophobic solutes, which have protein-like functional groups, solvation free energy approximation is better when the volume of the solute is used instead of the surface area [51,52]. Moreover, it was found that—on the fine-grained energy scale necessary to predict the high-resolution structure of proteins and protein–ligand complexes—the correlation between $∆G_{vdW}$ and the ASA of the solute is poor [17,18]. Therefore, modeling $∆G_{vdW}$ and $∆G_{cav}$ independently is better for accurate representation. Levy et al. also proposed the backbone of the AGBNP model [16,17]. Since $∆G_{vdW}$ and $∆G_{cav}$ are defined separately in the literature, $∆G_{cav}$ can be described as [22,53,54,55,56,57,58,59]:

(9) $$∆G_{cav}=\gamma ASA,$$

where γ represents surface tension coefficient.

Levy et al. [16] introduced more specific parameters and definitions for this model:

(10) $$∆G_{cav}=\sum_{i}\gamma_{i}A_{i}$$

where γ defines surface tension parameters of the corresponding atom, $A_{i}$ denotes the van der Waals surface area of the corresponding atom, and the summation is over solute atoms. However, in this model, instead of the free energy of cavity formation based on ASA, another approach based on van der Waals surface area and surface tension parameters was used [16,17,20]. They also described the solute–solvent van der Waals free energy, $∆G_{vdW}$, by an expression which was obtained as an integral of the van der Waals solute–solvent interactions over the solvent volume modeled as a uniform continuum [16,17,20]:

(11) $$∆G_{vdW}=\sum_{i}\alpha_{i}\frac{a_{i}}{{\left(B_{i}+R_{w}\right)}^{3}}\;$$

where $B_{i}$ is the Born radius of i’th atom and $R_{w}$ is the radius of the water molecule, $\alpha_{i}$, which is a scaling factor and is given by [16,17,20]:

(12) $$\alpha_{i}=-\frac{16}{3}\pi \rho_{w}\varepsilon_{w}\sigma_{iw}^{6}$$

$\rho_{w}$ is the density of the water molecule, and $\sigma_{iw}$ and $\varepsilon_{w}$ are the parameters of the OPLS force field [20,21].

As previously mentioned, the original AGBNP model relied solely on the van der Waals surface, calculated using the Poincaré formula [60], to estimate the solute’s surface area and volume [16,17]. This approach could lead to inaccurate approximations of Born radii and, consequently, did not adequately represent the exposed surface area in the model. To address these shortcomings, Levy et al. developed the AGBNP2 model, introducing a new term: the hydrogen bonding correction. The total solvation free energy in AGBNP2 is given by [17]:

(13) $$G_{solv}=∆G_{cav}+∆G_{ele}+∆G_{vdW}+∆G_{hb},$$

where $∆G_{hb}$ equals

(14) $$∆G_{hb}=\sum_{s}h_{s}S\left(w_{s};w_{a},w_{b}\right)$$

where $h_{s}$ is the maximum correction energy, which depends on the type of solute–water hydrogen bond, and $S(w_{s};w_{a},w_{b})$ is a polynomial switching function, which is found in detail in Ref. [17].

Later, it was proposed that nonpolar solvation ($∆G_{np})$ can be defined with repulsive free energy $\left(∆G_{rep}\right)$ and attractive free energy $\left(∆G_{att}\right)$ sub-components [18]. The nonpolar repulsive free energy ($∆G_{rep})$ can be modeled as [18]:

(15) $$∆G_{rep}=\;\gamma ASA+c\;$$

However, it has been observed that solvation free energy approximation depends on the usage of solvent-accessible volume or area. The applicability of ASA for describing hydrophobic solvation is limited to larger length scales; for surfaces with significant roughness or high curvature, solvent-accessible volume (SAV) serves as a more appropriate metric. It has been shown that for small cavities, repulsive free energy $\left(∆G_{rep}\right)$ correlates with the volume of the cavity, whereas for large cavities, it correlates with the cavity surface [18,19]:

(16) $$∆G_{rep}=\;pSAV+c\;$$

where SAV refers to solvent-accessible volume and p is a solvent pressure parameter.

On the other hand, Chandler et al. [18,22,23] showed that the attractive free energy $\left(∆G_{att}\right)$ can be described as [18,22,23]:

(17) $$∆G_{att}={<U}_{att}^{uv}>$$

where ${<U}_{att}^{uv}>$ is the van der Waals attractive interaction potential energy between solute u and solvent v and is described as the solvent-occupied volume integration [18]:

(18) $$<U_{att}^{uv}>=\sum_{\alpha =1}^{N_{s}}\int \rho_{aw}\left(r_{aw}\right)V_{att}(r_{aw})dr_{aw}$$

where $N_{s}$ is the sum over all solute atoms and $\rho_{aw}\left(r_{aw}\right)$ is a solvent distribution function around the solute atom a at a given solute–solvent distance, $r_{aw}$; $V_{att}\left(r_{aw}\right)$ is the attractive van der Waals potential in a decomposition scheme. However, due to the impossibility of knowing the value for $\rho_{aw}\left(r_{aw}\right)$ without equilibrium simulations in explicit solvent, it must be approximated [18].

### 2.2. Poisson–Boltzmann Equation

As mentioned above, solvation free energy is acquainted with electrostatic properties, $∆G_{ele}$, that have to be calculated. It was proposed that it can be calculated by solving the Poisson–Boltzmann equation with the continuum dielectric model [18,50,61]. The Poisson–Boltzmann equation is a widely used equation to describe the behavior of charged molecules in ionic solutions [10,62,63,64,65]. This equation allows us to model electrostatic interactions by combining the principles of the Poisson equation and Boltzmann distribution [9,10,62,63,64,65]. Initial attempts to solve it involved basic geometric models such as spheres for proteins [66] and cylinders for DNA [67]. Later, more accurate representations of the molecular surface were developed [68], such as the Solvent Excluded Surface [69], the Minimal Molecular Surface [70], and, more recently, the promising Blobby Surfaces. The latter utilizes a multilevel Gaussian density map [71]. Nevertheless, the Poisson equation relates the scalar electric potential (Φ) to the charge density (*ρ*) [9]. The Poisson–Boltzmann equation can be expressed as follows [9,72]:

(19) $$\vec{\nabla }\cdot \left[\varepsilon \left(r\right)\vec{\nabla }\Phi \left(r\right)\right]=-\rho_{B}\left(r\right)-\sum_{i}c_{i}^{\infty }z_{i}qe^{-\frac{z_{i}q\Phi \left(r\right)}{k_{B}T}}$$

where ε is the local dielectric constant, $c_{i}^{\infty }$ is the bulk concentration of the i’th ionic species, $z_{i}$ is the valence, and q is the unit charge. In such cases, when the exponential term is sufficiently small, it can be approximated by linearization. This leads to the formulation of the linear Poisson–Boltzmann equation:

(20) $$\vec{\nabla }\cdot \left[\varepsilon \left(r\right)\vec{\nabla }\Phi \left(r\right)\right]=-\rho_{B}\left(r\right)-\sum_{i}c_{i}^{\infty }z_{i}^{2}q^{2}\cdot \frac{q^{2}\Phi \left(r\right)}{k_{B}T}$$

Notably, the Poisson–Boltzmann equation and its discretization methods are explained in detail in the literature [73,74]. These are used to determine electrostatic free energy and can be shown as a function of the electrostatic part of the solvation free energy [75]:

(21) $$∆G_{ele}=\frac{1}{2}\int \Phi_{reac}\left(r\right)\rho \left(r\right)dV$$

where $\Phi_{reac}$ is the reaction field, which is the difference between the potentials that describe the solvent ${(\Phi }_{sol})\;$ and the vacuum ${(\Phi }_{vac})$.

Electrostatic free energy can also be decomposed into individual components. For example, Honig et al. provided a detailed description of this decomposition in their studies, which will be briefly summarized here [37,61,76]:

(22) $$∆G_{ele}=∆G_{ele}^{fixed}+∆G_{ele}^{ions}+∆G_{ele}^{sol}$$

where $∆G_{ele}^{fixed}$ is the fixed charge of solute and we obtain the following:

$$\Delta G_{ele}^{fixed}=\frac{1}{2}\int_{V}\rho^{\mathrm{fixed}}\left(r\right)\varphi \left(r\right)dv$$

$$\Delta G_{ele}^{ions}=k_{B}T\int_{V}\sum_{i}\left[c_{i}^{\infty }-c_{i}\left(r\right)\right]dv$$

$$\Delta G_{ele}^{sol}=-\frac{1}{2}\int_{V}\rho^{\mathrm{sol}}\left(r\right)\varphi \left(r\right)dv$$

Later, Nguyen et al. [77] showed that electrostatic solvation free energy $\left(∆G_{ele}\right)$ can be calculated with the Poisson–Boltzmann equation, such that

(23) $$∆G_{ele}=\frac{1}{2}\sum_{i=1}^{N_{m}}q_{i}(\Phi \left(r_{i}\right)-\Phi_{0}\left(r_{i}\right))$$

Here, Φ(r_i_) is the electrostatic potential at the position vector of the *i*th particle, $\Phi_{0}$ is the solution of the Poisson–Boltzmann equation without accounting for the solvent–solute interface, and qiis is the partial charge of the ith atom located at position $r_{i}$.

Additionally, the solution of Poisson–Boltzmann can also be obtained by the boundary element (BE) method, which can be found in detail in the literature. The BE method has some advantages, since it works directly with the polarization surface change density, and presents a numerical solution over the dielectric boundary. Since the molecular surface and the distribution of the surface BEs are determined by the distances between atoms, the BE method is unaffected by translations and rotations of molecules [78]. In this approach, the solute region and solvent region are separated by molecular surface S, and PB is transformed into an integral equation. The electrostatic potential is therefore given as follows:

(24) $$\Phi_{total}=\Phi_{mol}\left(r\right)+\Phi_{r}\left(r\right)$$

where $\Phi_{mol}$ denotes potential due to the solute charge distribution and $\Phi_{r}$ denotes the reaction potential due to the surface polarization density σ(s) on the molecular surface. Also, $\Phi_{r}$ can be defined as follows:

(25) $$\Phi_{r}\left(r\right)=\int_{S}\frac{\sigma \left(s\right)ds}{\left|r-s\right|}$$

The discretized version of integral equation of surface polarization density $\sigma \left(s\right)$ can be found in detail in Ref. [78] as a matrix form:

(26) σ=σK+b

The elements of matrix K denote the normal component of the electrostatic field on a boundary element, which is produced by a charged boundary element. The second term b denotes the electrostatic field generated by the charges of the solute molecule, which forms a permanent-source electrostatic field. Vorobjev et al. proposed an adaptive multigrid boundary element (MBE) to provide the fast calculation of the electrostatic field in the multigrid representation of the dielectric border surface [78]. In order to solve the matrix equation and to decrease the computational weight of variables, they defined three sets of BE on the molecular surface, i.e., small, large, and patch BEs. The polarization charge densities, however, are constant over respective BEs. Each charged atom or group of atoms is considered separately, as detailed in the work of Vorobjev et al. The total polarization charge density of the whole molecule therefore is defined as follows:

(27) $$\sigma \left(s\right)=\sum_{k}\sigma_{k}\left(s\right)$$

where $\sigma_{k}$ denotes the polarization charge density due to charged atom k. Additionally, the macromolecular surface can be divided into three regions. These are local surface $S_{loc}$, represented by small BEs; intermediate surface $S_{Int}$, represented by large BEs; distant surface $S_{Dst}$, represented by patch BEs. Also, polarization charge density distribution, therefore, can be approximated with low-dimension vectors, $\sigma^{L},\;\sigma^{I},\sigma^{D}$ and the source term, therefore, is $b^{L},b^{I},b^{D}$. This leads to the representation of the matrix form as a linear equation involving low vectors:

(28) $$\left(\begin{array}{c} \sigma^{L}\\ \sigma^{I}\\ \sigma^{D} \end{array}\right)=\left(\begin{array}{ccc} K^{LL} & K^{LI} & K^{LD}\\ K^{IL} & K^{II} & K^{ID}\\ K^{DL} & K^{DI} & K^{DD} \end{array}\right)\left(\begin{array}{c} \sigma^{L}\\ \sigma^{I}\\ \sigma^{D} \end{array}\right)+\left(\begin{array}{c} b^{L}\\ b^{I}\\ b^{D} \end{array}\right)$$

where $K^{LL}$, $K^{IL}$, $K^{DL}$, $K^{IL}$, $K^{II}$, $K^{ID}$, $K^{DL}$, $K^{DI}$, and $K^{DI}$ are corresponding matrix elements. Briefly, MBE works with three levels, and it can be summarized as such. Firstly, a set of small and large BEs on the molecular surface are calculated and those BEs are collected into the patch Bes; then, the centers of molecular electrostatic fields are defined as a set of charged atoms or a compact atom group. For each electrostatic field center, corresponding MBE sets are calculated. After that, the matrix elements calculated and the matrix is solved using the preconditioned biconjugate gradient iterative method [78]. Lastly, polarization charge densities are collected on the MBE, and this process is repeated for each electrostatic field center until everything is solved.

Notably, several Gaussian-based analytical models have been proposed [79,80]. For instance, in the work of Scheraga et al., the exposed volume of hydration (VHS) is explained. Since the hydration interaction free energy is proportional to its exposure to water, it can be expressed as

(29) $$∆G_{hyd}=\sum_{i}\delta_{i}{\left(VHS\right)}_{i}$$

where $\delta_{i}$ is empirical free energy of hydration density for atom i, and ${\left(VHS\right)}_{i}$ denotes volume of the hydration shell for atom i, which is exposed to water. Also, proteins consisted of multiple atoms; therefore, the collection of overlapping corresponding spheres volume can be expressed briefly as

(30) $$V=\sum_{i}S_{i}-\sum_{ij}D_{ij}+\sum_{ijk}T_{ijk}-\sum_{ijkl}Q_{ijkl}+\cdots$$

where S represents the volume of a single sphere and D, T, and Q represent the volume of the intersection of two, three, and four spheres, which can be further extended; however, it is incompatible with the Fourier–Poisson integral. Additionally, reducing overlaps could lead to large errors. To address this, an artificial reduction in the van der Waals radii of all atoms other than atom i is employed when calculating ${\left(VHS\right)}_{i}$:

(31) $$\;{\left(VHS\right)}_{i}=\frac{4\pi }{3}\left({\left(R_{i}^{h}\right)}^{3}-{\left(R_{i}^{v}\right)}^{3}\right)-\sum_{j\neq i}\left[D\left(r_{ij};R_{i}^{h},R_{j}^{r}\right)-D\left(r_{ij};R_{i}^{v},R_{j}^{r}\right)\right]$$

where $R_{i}^{h}$ denotes the radius of the first hydration shell of atom i, $R_{i}^{v}$ is its van der Waals radius, and $D\left(r_{ij};R_{i},R_{j}\right)$ denotes the volume of intersection of two spheres of radii $R_{i}$ and $R_{j},$ whose centers are separated by $r_{ij}$. $R_{j}$ is the reduced van der Waals radius for atom j.

The volume of the intersection of two spheres solved via a Gaussian function, explained in detail in the Ref. [79], thus yields the formula for the volume of the intersection of two Gaussian spheres:

(32) $$D\left(r_{12};R_{1},R_{2}\right)=A^{2}{\left(\frac{\pi B}{R_{1}^{2}+R_{2}^{2}}\right)}^{\frac{3}{2}}R_{1}^{3}R_{2}^{3}e^{\left(\frac{-r_{12}^{2}}{B\left(R_{1}^{2}+R_{2}^{2}\right)}\right)}$$

where $R_{1}$ and $R_{2}$ are the hard-sphere radii and $r_{12}$ denotes the separation between the centers of the spheres and A and B are scaling parameters.

Makowski et al. proposed a Gaussian differential overlap-based model for the energetics of hydrophobic association by analyzing the number and context of water molecules in different parts of the hydration sphere of hydrophobic solute pairs, with solute and solvent densities approximated as the Gaussian matching dimension and the symmetry of the solute [80]. The potential mean force can be described as follows:

(33) $$W=E_{vdW}+E_{ele}+∆F_{cav}$$

where W denotes the potential mean force in general; $E_{vdW}$ is the van der Waals term; $∆F_{cav}$ denotes the difference between the cavity contribution to the free energy of the hydration of the dimer and that of the isolated monomer; $E_{ele}$ collectively denotes electrostatic interactions, polarization energy between solute particles, and the contribution of solvent polarization to energy, as explicitly explained in Ref. [80]. Moreover, $∆F_{cav}$ based on the hydration shell volume and Gaussian overlap is used to express the change in the free energy of hydration:

(34) $$∆F_{cav}=\frac{\alpha_{ij}^{\left(1\right)}x^{\frac{1}{2}}\;+\alpha_{ij}^{\left(2\right)}x-\alpha_{ij}^{\left(3\right)}}{1+\alpha_{ij}^{\left(4\right)}+x^{12}}$$

where $\alpha_{ij}^{\left(n\right)}$, n=1,2,3… are the constants of the expression and $r_{ji}$ denotes the distance between the centers of the interacting sites; x denotes $x=r_{ij}\sqrt{\sigma_{i}^{2}+\sigma_{j}^{2}},$ where $\sigma_{i}$ is the solvation radius of particle i.

They also introduced the formulation of the solvent and solute density as spheroidal Gaussians, as at that time in the United Residue (UNRES) Force Field, the side chains were treated as ellipsoids; therefore, $∆F_{cav}$ can be expressed as follows [81]:

(35) $$∆F_{cav}=\frac{\alpha^{\left(1\right)}\left[{\left(x\lambda \right)}^{\frac{1}{2}}+\alpha^{\left(2\right)}x\lambda -\alpha^{\left(3\right)}\right]}{1+\alpha^{\left(4\right)}{\left(x\lambda \right)}^{12}}$$

where

$$x=\frac{d}{\sqrt{\sigma_{1}^{2}+\sigma_{2}^{2}}}$$

and

$$\lambda ={\left[1-\frac{\chi_{1}\omega_{1}^{2}+\chi_{2}\omega_{2}^{2}-{2\chi }_{1}\chi_{2}\omega_{1}\omega_{2}\omega_{12}}{1-\chi_{1}\chi_{2}\omega_{12}^{2}}\right]}^{\frac{1}{2}}$$

with,

$$\omega_{1}={\hat{u}}_{1}{\cdot \hat{r}}_{12},\;\omega_{2}={\hat{u}}_{2}{\cdot \hat{r}}_{12},\;\omega_{12}={\hat{u}}_{1}\cdot {\hat{u}}_{2},$$

$$\chi_{1}=\frac{\sigma_{∥1}^{2}-\sigma_{⊥1}^{2}}{\sigma_{∥1}^{2}+\sigma_{⊥2}^{2}}$$

and

$$\chi_{2}=\frac{\sigma_{∥2}^{2}-\sigma_{⊥2}^{2}}{\sigma_{∥2}^{2}+\sigma_{⊥1}^{2}}$$

where d denotes the distance between the centers of the particles, ${\hat{u}}_{1}$ and ${\hat{u}}_{2},$ respectively, denote the unit vector along the long axis of the first particle and second particle and ${\hat{r}}_{12}$ is the unit vector along the vector pointing from the first to the second particle. $\alpha^{\left(n\right)},n=1,2,3\ldots$ are coefficients used to represent the combination of the different contributions of different hydration regions, which are explained in detail in Ref. [81]. Lastly, $\sigma_{∥}$ and $\sigma_{⊥},$ respectively, are the dimensions of the long and the two short axes of the ellipsoid.

### 2.3. Born Equation

The Born equation describes the transfer free energy of a spherical ion from the gas phase to a dielectric medium, where both the potential and solvation free energy are determined based on [11,37,75,82]:

(36) $$\Delta G_{ele}=-\frac{q^{2}}{2a}\left(1-\frac{1}{\varepsilon_{w}}\right)\;$$

where a is the ion radius, q is its charge, and $\varepsilon_{w}$ is the continuum dielectric, which represents water. It can also be generalized as [9,82]:

(37) $$\Delta G_{ele,i}=-\frac{q_{i}^{2}}{R_{i}}\left(\frac{1}{\varepsilon_{in}}-\frac{1}{\varepsilon_{out}}\right)$$

where $R_{i}$ represents the Born radii.

The electrostatic solvation free energy, depending on the Green function via the Generalized Born model, exists in different forms in the literature [37,50]. The electrostatic free energy part of the solvation free energy can be formulated as [11,37]:

(38) $$∆G_{ele}=-\frac{1}{2}\left(1-\frac{1}{\varepsilon_{w}}\right)\sum_{i,j\;}^{N}\frac{q_{i}q_{j}}{f_{GB}}\;$$

where $f_{GB}$ is a function that relates the effective Born radii through the work in [83]. It has been defined in the literature [9,11,37,50,75,84]. Notably, one reliable Generalized Born formula was proposed by Still et al. [85]. Sigalov et al. [82] expanded the formulation beyond the canonical form of the Green function:

(39) $$∆G_{ele}=-\frac{1}{2}\left(\frac{1}{\epsilon_{in}}-\frac{1}{\epsilon_{out}}\right)\frac{1}{1+\beta \alpha }\sum_{ij}q_{i}q_{j}\left(\frac{1}{f^{GB}}+\frac{\alpha \beta }{A}\right)\;$$

Here, β is the ratio of the dielectric constants, *A* is the electrostatic size of the considered molecule, and α is a constant parameter.

Lazardis et al. proposed another model (EEF1), which is a less heuristic approach and combines an excluded volume approach with a modified version of the polar hydrogen energy function [86]. Since the solvation free energy, $∆G_{solv},$ of a given macromolecular conformation can be written as an integral over the space around it, we obtain

(40) $$∆G_{solv}=\sum_{i}∆G_{solv}^{i}$$

Moreover, it can be represented as an integral over the space around it:

(41) $$∆G_{solv}=\sum_{i}\int f\left(r\right)dr$$

$f\left(r\right)$ is the solvation free energy density at point r. This can be further improved by treating the solute–solvent interaction energy as the sum of group–solvent interactions and representing solute–solvent correlation function as a product of group–solvent correlation functions:

(42) $$∆G_{solv}^{i}=∆G_{ref}^{i}-\sum_{j}\int_{Vj}f_{i}\left(r\right)dr$$

This can be decomposed into a sum of pairwise interaction:

(43) $$∆G_{solv}=\sum_{i}∆G_{solv}^{i}=\sum_{i}∆G_{ref}^{i}-\sum_{i}\sum_{i\neq j\;}f_{i}\left(r_{ij}\right)V_{j}\;\;\;\;$$

Here, $∆G_{ref}^{i}$ refers to the reference solvation free energy in which i is essentially fully solvent-exposed group [86]:

(44) $$\sum_{i}∆G_{ref}^{i}\sum_{j>i\;}{(f}_{i}\left(r_{ij}\right)V_{j}+f_{j}\left(r_{ij}\right)V_{i})\;\;$$

$f_{i}(r)$ is the solvation free energy density of the group i at point r. It consists of solute–solvent energy, solvent reorganization energy, solute–solvent entropy, and solvent reorganization entropy. It is a function that rapidly changes with distance, where it has large values near the solute but these vanish at increasing distances. A typical form would be a Gaussian type function. Thus,

(45) $$\;∆G_{solv}=\sum_{i}∆G_{ref}^{i}-\sum_{j>i}\left[\frac{2∆G_{free}^{i}}{4\pi \sqrt{\pi }\lambda_{i}r_{ij}^{2}}e^{-x_{ij}^{2}V_{j}}+\frac{2∆G_{free}^{j}}{4\pi \sqrt{\pi }\lambda_{j}r_{ij}^{2}}e^{-x_{ji}^{2}V_{i}}\right],$$

where $∆G_{free}^{i}$ refers to the solvation free energy of the isolated group and $\lambda_{i}$ is a correlation length [86].

### 2.4. The DelPhI Model

Developed in the early 1990s and acquiring many updates since then, DelPhI is designed to solve the Poisson–Boltzmann equation for biomolecular electrostatics and it is still widely used [68,87,88,89]. Due to its continuous expansion, we will introduce its fundamentals. Here, we introduce a version of the Poisson–Boltzmann equation:

(46) $$\vec{\nabla }\cdot \left[\varepsilon \left(r\right)\vec{\nabla }\Phi \left(r\right)\right]-\left[K{\left(x\right)}^{2}\right]\sinh\left(\Phi \left(x\right)\right)=-4\pi \rho \left(x\right)$$

where $\Phi \left(x\right)$ is the electrostatic potential and it is determined by $\varepsilon \left(x\right)$. The spatial dielectric function, $K{\left(x\right)}^{2}$, is a modified Debye–Hückel parameter, and the reason for its use is the dielectric discontinuity resulting from the difference in the electronic polarizability between the macromolecule and the surrounding solvent, which significantly affects electrostatic interactions. Lastly, ρ(x) is the charge distribution function. Linearization is carried out by approximating the hyperbolic sine function using its argument, effectively replacing the nonlinear sinh term with a linear expression [87].

They also considered cubic lattices, in which each side contained L grid points, for a total of $N=L^{3}$ points. Thus, the final reduction to finite difference form yields

(47) $$\Phi_{0}=\left[\frac{\left(\sum_{i=1}^{6}\varepsilon_{i}\Phi_{i}\right)+4\pi q_{0}/h}{\left(\sum_{i=1}^{6}\varepsilon_{i}\right)+{\left(K_{0}h\right)}^{2}}\right]$$

where $\Phi_{0}$ denotes the potential at a particular grid point, $\Phi_{i}$ is the potential at the six nearest neighbors, and $\varepsilon_{i}\;is$ the dielectric constant at the midpoint between $\Phi_{0}$ and $\Phi_{i}$. The charge assigned to the grid point is given by $q_{0}$, and $K_{0}$ is nonzero if the grid point is in salt. The parameter h denotes the grid spacing, which is given in Ångstroms [81]. As such, $\Phi_{i}$ can be mapped, in general, as

(48) $$\Phi \left(x,y,z\right)=\Phi_{i}\;\mathrm{and}\;i=g(x,y,z)$$

where g(x,y,z) is an arbitrary mapping function.

Also, it can be written as a matrix:

(49) Φ=TΦ+Q

where T denotes a matrix, and Φ and Q are vectors. The elements of the matrix are as follows:

(50) $${\;T}_{ij}=0\;if\;j\neq g\left(x\pm 1,y,z\right),g\left(x,y\pm 1,z\right),g\left(x,y,z\pm 1\right)$$

The elements of Q are as follows:

$$Q_{i}=[4\pi q(x,y,z)/h]*d(x,y,z)$$

where d(x,y,z) can be discretized as

(51) $$\;d\left(x,y,z\right)=1/\;[\;\varepsilon \left(x,\;x\pm 1,y,z\right)+\varepsilon \left(x,y,y\pm 1,z\right)++\varepsilon \left(x,y,z,z\pm 1\right)+{\left(K_{0}h\right)}^{2}]$$

However, boundary conditions are crucial and are typically fixed by using Coulomb’s law or Debye–Hückel theory. At a sufficient distance from the molecular surface, the potential becomes insensitive to the specific molecular shape, thus making the choice of box fill ratio important [87].

Additionally, the Jacobian relaxation method is prone to slow convergence as the grid increases, thus making large-scale computations costly in terms of both time and memory. To address these issues, Gauss–Siedel relaxation can be employed, which uses newly updated potential values during the iteration process. Unlike the Jacobian method, where the potential at a current iteration is calculated solely based on the potential of the previous iteration, Gauss–Seidel relaxation incorporates recent potential values into the ongoing iteration. However, the implementation order of grid points can vary; thus, the spectral radius and intrinsic rate of convergence of the method remain independent of this mapping. Due to nature of the matrix, which is consistently ordered, for the Poisson–Boltzmann problem, Gauss–Seidel converges approximately twice as fast as the Jacobian method, requiring only half as many iterations [87], which leads to the spectral radius of Gauss–Seidel being the square of that for Jacobian method:

(52) $$\lambda_{N}\left(Seidel\right)={\left[\lambda_{N}(Jacobian)\right]}^{2}$$

This is explained by the checkerboard ordering; thus, the matrix, T, can be denoted as

(53) $$T=\left[\begin{array}{cc} 0 & T_{1}\\ T_{2} & 0 \end{array}\right]$$

$T_{1}$ updates entries with odd entries, and $T_{2}$ updates odd entries with even entries. However, Successive Over-Relaxation significantly speeds up Gauss–Siedel calculations to solve the Poisson–Boltzmann equation [87], and the optimal value of the relaxation parameter is expressed as ω=1/(1−α):

(54) $$\omega =\left(2/(1+\sqrt{1-\lambda_{N}})\right)$$

The minimum spectral radius is:

(55) $$\lambda_{N}=\left(\lambda_{N}^{gs}/{\left[1+\sqrt{1-\lambda_{N}^{gs}}\right]}^{2}\right)$$

$\lambda_{N}^{gs}$ denotes spectral radius of Gauss–Seidel. With the substitution, $\varepsilon =1-\omega_{N}^{gs}$, the spectral radius is as follows

(56) $$\lambda_{N}=\frac{1-\varepsilon }{{\left(1+\sqrt{\varepsilon }\right)}^{2}}\approx 1+2\varepsilon -2\sqrt{\varepsilon }$$

and since ε is a small number, $\sqrt{\varepsilon }$ is significantly larger. This denotes a significant increase in the rate of convergence. However, the spectral radius is sensitive to ω, and the $\lambda_{N}$ is chosen poorly, thus all improvements are lost; therefore, Honig et al. introduced optimal Successive Over-Relaxation [87], which reduced the number of iterations by several orders of magnitude to a few iterations. However, using $\lambda_{N}$ from a few iterations, based on eigenvalue problems to find the maximum eigenvalue T is vital. This can be performed by acquiring the minimum eigenvalue of 1−T. This is related to Connected-Moment Expansion, which gives an approximation of the ground state wavefunction [87]. After approximation of the ground state wavefunction and Hamiltonian, vector Q is set to zero:

(57) $$\Phi \left(n\right)=T^{n}\Phi \left(0\right)$$

Thus, the prescription is to set the initial vector equal to the highest eigenvalue eigenstate approximation, set all charges equal to zero, and calculate for a few moments [87].

Later, in order to better expression for electrostatic free energy, the authors implemented a more accurate definition in DelPhI [88], meaning that free energies became independent of the lattice used to solve the PB equation. The energy density can be expressed as

(58) $$∆g=\frac{1}{2}\rho_{fixed}\Phi -\sum_{i}∆\pi_{i}-\frac{1}{2}\;\rho_{solv}\Phi$$

Additionally, in the finite-difference method, the system is discretized. The term $\frac{1}{2}\rho_{fixed}\Phi$, can also be expressed as

(59) $$\frac{1}{2}\rho_{fixed}\Phi =\frac{1}{2}\sum_{j}q_{j}\rho \left({\vec{r}}_{j}\right)$$

where the electrostatic potential is calculated from all sources of charge, except the one positioned at ${\vec{r}}_{j}$ [87]. This method eliminates accuracy concerns by representing the potential at a specific point as a superposition of real charges, surface polarization effects, and contributions from mobile ions,; therefore, the potential at the position of charge j can be written as

(60) $$\Phi \left({\vec{r}}_{j}\right)=\Phi_{coul}\left({\vec{r}}_{j}\right)+\Phi_{reaction}\left({\vec{r}}_{j}\right)+\Phi_{ion}\left({\vec{r}}_{j}\right)$$

where Coulombic potential, generated by other fixed charges, can be expressed as

(61) $$\Phi_{coul}\left({\vec{r}}_{j}\right)=\sum_{i\neq j}\frac{q_{i}}{4\pi \epsilon_{0}\epsilon_{i}\left|{\vec{r}}_{i}-{\vec{r}}_{j}\right|}$$

The corrected reaction field term, arising from the polarization of the boundary between different media, is

(62) $$\Phi_{reaction}\left({\vec{r}}_{j}\right)=\sum_{i\neq j}\frac{\delta_{p}}{4\pi \epsilon_{0}\epsilon_{i}\left|{\vec{r}}_{i}-{\vec{r}}_{j}\right|}$$

where p denotes the positions of surface polarization charges, and $\delta_{p}$ represents the magnitude of the polarization charge at location $r_{p}$ on the surface. These charges are determined based on Gauss’s law and the electrostatic potential map generated by the finite-difference method [82,83]. The last term, potential generated by mobile ions in solution, is defined as follows:

(63) $$\Phi_{ion}\left({\vec{r}}_{j}\right)=\sum_{k}\frac{h^{3}\delta_{p_{k}^{ion}}}{4\pi \epsilon_{0}\epsilon_{solv}\left(r_{j}-r_{k}\right)}$$

where h denotes grid spacing, and $\delta_{p_{k}^{ion}}$ denotes the net ion charge density at grid point k in solution, where k denotes grid points in the solution [88].

### 2.5. The APBS Model

The solution of the Poisson–Boltzmann equation for biomolecules however remained a challenge. Since the dielectric value of dielectric permittivity jumps drastically at the interface between solvent and solution, the function of the ionic strength of the solvent is discontinuous at the surrounding region of the biomolecule, and the formula itself features delta functions and rapid nonlinearity that causes difficulties in analytical approaches. Different approaches have been proposed to approximate and calculate the Poisson–Boltzmann equation [90,91,92], in which some of them include uniform-mesh finite approaches [90,93], integral-based methods [94,95], and nonadaptive finite element methods [92,96].

Here, we show a slightly different version of Poisson–Boltzmann equation for the 1:1 electrolyte:

(64) $$\;-\vec{\nabla }\cdot \left(\epsilon \left(x\right)\vec{\nabla }u\left(x\right)\right)+{\bar{K}}^{2}\left(x\right)\sinh\left(u\left(x\right)\right)\;=\frac{4\pi e_{c}^{2}}{k_{B}T}\sum_{i=1}^{N_{m}}z_{i}\delta (x-x_{i})$$

where ${\bar{K}}^{2}$ is Debye–Hückel parameter, $N_{m}$ is the total number of point charges, and $\delta (x-x_{i})$ is the delta function that reflects point-charge behavior of the charge at $x_{i}$ [97,98].

To address the challenges, Holst et al. developed the Adaptive Poisson–Boltzmann Solver (APBS) model, where they adopted an adaptive multilevel finite element method to solve the nonlinear Poisson–Boltzmann equation, in which a posteriori error estimation guides the refinement [97,98]. The abovementioned Poisson–Boltzmann equation is valid if the solution function is differentiable twice, which is generally not satisfied in biomolecular systems. Therefore, a weak formulation is required for the finite element method due to obtaining reduced differentiability:

(65) $$\langle F\left(u\right),v\rangle =\int_{\Omega }\left[\left(\epsilon \nabla u\right)\nabla v+{\bar{K}}^{2}\left(x\right)\sinh\left(u\right)v-fv\;\right]dx$$

where v is the test function from Sobolev Space, $H_{0}^{1}(\Omega )$, and $f\left(x\right)$ represents the charge distribution term. In order to employ the Galerkin method, an approximation ${\bar{u}}_{h}$ to the boundary potential function $\bar{u}$ is constructed, and to satisfy Poisson–Boltzmann Equation, it is necessary to solve $u_{h}\in {\bar{u}}_{h}+V_{h},$ such that

(66) $$F\left(u_{h}\right),\;v_{h}=0,\;\forall v_{h}\in V_{h}$$

where $V_{h}$ is subspace of the Sobolev Space. Additionally, the Poisson–Boltzmann Equation is

(67) $$-\vec{\nabla }\cdot \left(a\left(x\right)\vec{\nabla }u\left(x\right)\right)+b\left(x,u\left(x\right)\right)=0\;in\;\Omega ,$$

(68) $$u\left(x\right)=g\left(x\right)on\;\partial \Omega ,$$

where a denotes $a:\Omega ⟼R^{3\times 3},b:\Omega \;x\;R⟼R,and\;g:\Omega \;⟼\partial R$ [93].

In order to apply a Newton iteration, linearization is required [97]. To linearize the Poisson–Boltzmann equation operator, $\langle F\left(u\right),v\rangle$, a bilinear linearization form $\langle DF\left(u\right)w,v\rangle$ is produced:

(69) $$\langle DF\left(u\right)w,v\rangle ={\left.\frac{d}{dt}\;\langle F\left(u+tw\right),v\rangle \right|}_{t=0}$$

and it follows,

(70) $$=\int_{\Omega }\;(a\vec{\nabla }w\cdot \vec{\nabla }v+\frac{\partial b\left(x,u\right)}{\partial u}wv)dx$$

where u, v, and w are three arguments of the scalar-valued function. $\langle F\left(u\right),v\rangle$, and the associated bilinear linearization form, $\langle DF\left(u\right)w,v\rangle$ together with a continuous piecewise polynomial subspace of the solution space, ${\bar{u}+H}_{0}^{1}(\Omega )$, are required to employ the finite element method for the numerical solution of the original elliptic equation [97,98].

Moreover, the inexact Newton method provides a numerical solution to the nonlinear Poisson–Boltzmann equation by iteratively solving its linearized versions to calculate adjustments until a desired error tolerance is met, where the computationally intensive part of this method is the complexity of the linear algebraic equations generated in each step. Notably, multilevel methods are near-optimal techniques for solving such equations resulting from the discretization of a large class of general linear elliptic problems [97,98]. These methods operate by using an interpolation operator to map the discretized linearized equation onto a coarser mesh, which reduces the number of variables and simplifies the problem. The approximate solution on the finest mesh can then be obtained by using the coarse solutions to accelerate the convergence of iterative methods on the finer levels [97,98].

Another aspect of the APBS is the posteriori error estimation and adaptive refinement, where the main idea can be understood by considering an approximation, $\tilde{u}$, to the solution of a linear equation, defined by a nonsingular operator A [98]:

(71) Au=f

The quality of the approximation can be tested by forming the residual. Since the error in the approximation $e\;=\;u-\;\tilde{u}$ satisfies the error equation,

(72) $$Ae=A\left(u-\tilde{u}\right)=Au-A\tilde{u}=f-A\;\tilde{u}=r$$

inverting A and taking norms of both sides thus gives:

(73) $$\left|\left|e\right|\right|=\left|\left|A^{-1}r\right|\right|\leq \left|\left|A^{-1}\right|\right|\cdot \left|\left|r\right|\right|$$

This inequality shows that the error in approximation is limited by the product of the inverse operator and residual norm. Thus, the estimation of the error of the solution can be obtained [98].

### 2.6. The ABSINTH Model

It has been noted that, due to EEF1′s incapabilities, other implicit solvent approaches have advantages such as reproducing the disordered nature of an amyloid fragment [99]. Intrinsically disordered proteins are widely present in the archaeaic, prokaryotic, and eukaryotic proteomes [100,101,102]. Due to the fact that implicit solvents are prone to fail to model these disordered structures, Pappu et al. introduced the self-assembly of biomolecules studied by an implicit, novel, and tunable Hamiltonian (ABSINTH) model [28]. Although it was related to the work of Lazardis et al. [86], it was developed to address the accurate modeling of intrinsically disordered proteins. The Hamiltonian of this model can be formulated as

(74) $$E_{total}=W_{Solv}+U_{LJ}+W_{ele}+U_{corr}$$

$W_{Solv}\;$ is the solvation term that corresponds to the direct mean field interaction, $U_{LJ}$ represents the dispersive and short-range steric interactions contributions, $U_{corr}$ defines the torsional correction terms applied to dihedral angles influenced by electronic effects which could not be captured by $U_{LJ}$, and $W_{el}$ represents electrostatic interactions modulated by the mean-field dielectric [28]. Since the degree of the solvent accessibility has influence on the direct mean field interactions, it can be formulated as

(75) $$W_{solv}=\sum_{i=i}^{N_{SG}}\zeta_{i}∆G_{solv}^{i}=\sum_{i=i}^{N_{SG}}[\sum_{k=1}^{n_{i}}\lambda_{k}^{i}v_{k}^{i}]\cdot ∆G_{solv}^{i}$$

Accordingly, it denotes the solvation free energy of solvation group i, while the average weighted energy over its possible states, $v_{k}^{i}$, is calculated using the weight factors $\lambda_{k}^{i}\;$ [28]. 

### 2.7. Quasi-Chemical Theory

Quasi-Chemical Theory can be summarized as a molecular-level framework for estimating the excess chemical potential of solutes, where the region around the solute of interest is partitioned into inner- and outer-shell domains [103,104,105]. For instance, it implies that the chemical potential of a lithium ion ${(µ}_{Li^{+}})$ can be expressed in terms of interaction of ideal and non-ideal contributions [104]:

(76) $$\;\beta^{*}\mu_{Li^{+}}=\log_{e}\left(\frac{\rho_{Li^{+}}}{q_{Li^{+}}/V}\right)\;-\;{log}_{e}\rho_{0}-\log_{e}\left(\sum_{n\geq 0}{Ǩ}_{n}\rho_{H_{2}O}^{n}\right),$$

where $\beta^{*}$ denotes $\frac{1}{RT}$. The ion hydration reactions can be modeled through inner-shell interactions without considering medium effects [104,106]:

$$Li^{+}+nH_{2}O\;⇌\;Li^{+}{\left(H_{2}O\right)}_{n}$$

In order to understand the Quasi-Chemical Theory, we need to describe the potential distribution theorem [104,107]:

(77) $$\;\rho_{\sigma }={\left\langle e^{-\beta^{*}∆U}\right\rangle }_{0}z_{\sigma }\left(\frac{q_{\sigma }}{V}\right)$$

$\rho_{\sigma }$ is the density of the considered molecule, $q_{\sigma }$ is the single-molecule partition function for that molecule, and $z_{\sigma }$ is the absolute activity of that molecule and equals to

$$z_{\sigma }=e^{\beta^{*}\mu_{\sigma }}$$

Asthagiri et al. studied the influence of chemical effects, which are based on inner-shell reactions [103]:

$$X^{\pm }+nH_{2}O\;⇌\;X{{\left(H_{2}O\right)}^{\pm }}_{n}$$

$X^{\pm }$ denotes various ions such as $H^{+},Li^{+},Na^{+},OH^{-}$ [103].

Furthermore, the basic quasi-chemical approximation of the excess chemical potential of an ion in water can be described as

(78) $$\;\beta *{\bar{\mu }}_{X\pm \left(w\right)\;}\approx -\ln\left({Ǩ}_{n}\rho_{H_{2}O}^{n}\right)$$

where β equals to $1/k_{B}T$ and differs from $\beta^{*}$ since it incorporates R. ${Ǩ}_{n}$ equals

$${{Ǩ}_{n}=Ǩ}_{n}^{\left(0\right)}*e^{-\beta \left({\bar{\mu }}_{X{\left(H_{2}O\right)}_{n}}^{\pm }\left(w\right)-{\bar{\mu }}_{H_{2}O}(w)\right)}$$

${Ǩ}_{n}^{\left(0\right)}$ refers to the equilibrium constant for the reaction in an ideal gas state, ${\bar{\mu }}_{X{\left(H_{2}O\right)}_{n}}^{\pm }\left(w\right)$ is the excess chemical potential of the ion–water cluster, and ${\bar{\mu }}_{H_{2}O}(w)$ is the excess chemical potential of a water molecule in bulk water.

### 2.8. Transfer Free Energy Approach

The (mis)folding of intrinsically disordered proteins remains a challenge [108]. Shea et al. introduced a more accurate implicit solvation model for such systems [29].

The model can be defined as

(79) $$G_{solv}=E_{vac}+G_{ele}+G_{np}$$

$E_{vac}$ represents the peptide energy in a vacuum which results from both internal bonded contributions such as angles and dihedrals and non-bonded van der Waals interactions. They also introduced a new approach where the surface tension coefficient had a dependency on temperature. In fact, the probability of an unburied side chain of residue i, $P_{nb}^{sc,i}$, can be calculated as

(80) $$P_{nb}^{sc,i}=\frac{1}{N_{i}}\sum_{k=1}^{N_{i}}\left[\frac{ASA_{k}^{sc,i}\;}{ASA_{max}^{sc,i}\;}\right]$$

$ASA_{k}^{sc,i}$ defines side chain solvent accessibility of the k’th residue type i and $ASA_{max}^{sc,i}$ refers to the maximum solvent accessibility of side chain type i  [30]. Based on this logic. the probability of the backbone can be calculated as

(81) $$P_{nb}^{bb}=\frac{1}{N_{i}}\sum_{k=1}^{N_{i}}\left[\frac{ASA_{k}^{bb}\;}{ASA_{max}^{bb}\;}\right]$$

Thus, the energy can be calculated as

(82) $$E_{nb}^{sc,\frac{i}{bb}}=-RTlnP_{nb}^{sc,\frac{i}{bb}}$$

The energy of buried side chain can be calculated using

(83) $$E_{b}^{sc,\frac{i}{bb}}=-RT\;\ln\left(1-P_{nb}^{sc,\frac{i}{bb}}\right)$$

These assessments can help in calculating the energetic cost for exposing side chain type i or the peptide backbone between two different temperatures via

(84) $$\;{\left(E_{nb}^{sc,\frac{i}{bb}}-E_{bb}^{sc,\frac{i}{bb}}\right)}_{T}-{\left(E_{nb}^{sc,\frac{i}{bb}}-E_{bb}^{sc,\frac{i}{bb}}\right)}_{T_{0}}=\left[\gamma_{2}^{sc,\frac{i}{bb}}\left(T\right)-\gamma_{0}\right]ASA_{k}^{sc,\frac{i}{bb}}$$

Nonpolar contribution can be expressed as

(85) $$\;{\;G}_{np}=\left\{\begin{array}{c} \gamma_{0}ASA,\;T=T_{0}\\ \gamma_{0}ASA+\sum_{k=1}^{n}\left(\gamma_{2}^{sc,k}\left(T\right)-\gamma_{0}\right)ASA^{sc,k}\;+\sum_{k=1}^{n}\left(\gamma_{2}^{bb}\left(T\right)-\gamma_{0}\right)ASA^{bb,k}\;,\;T\neq T_{0}\; \end{array}\right.$$

Fractional solvent accessibility can be described as

(86) $$G_{np}=\left\{\begin{array}{c} \gamma_{0}ASA,\;T=T_{0}\\ \gamma_{0}ASA+\sum_{k=1}^{n}∆g_{tr,2}^{sc,k}\left(T\right)\alpha^{sc,k}+∆g_{tr,2}^{bb}\left(T\right)\sum_{k=1}^{n}\alpha^{bb,k}\;,\;T\neq T_{0}\; \end{array}\right.$$

where $\alpha^{sc,k/bb,k}$ is a fractional solvent accessibility parameter found in Ref. [30] and $∆g_{tr,2}^{sc,k/bb,k}$ can be written as

$$∆g_{tr,2}^{sc,k/bb,k}=(\gamma_{2}^{sc,k/bb}(T)-\gamma_{0})ASA_{GLY-k-GLY}^{sc,k/bb,k}$$

The general formula can be written as

(87) $$G_{solv}=E_{vac}+G_{ele}+G_{np}^{0}+G_{tr}\left(T\right)$$

The second approach can be described as

(88) $$\;G_{tr}\left(T\right)=\left\{\begin{array}{c} 0,\;T=T_{0}\\ \gamma_{0}ASA+\sum_{k=1}^{n}∆g_{tr,2}^{sc,k}\left(T\right)\alpha^{sc,k}+∆g_{tr,2}^{bb}\left(T\right)\sum_{k=1}^{n}\alpha^{bb,k}\;,\;T\neq T_{0}\; \end{array}\right.$$

(89) $${G_{np}^{0}=\gamma }_{0}ASA$$

Shea et al. [29] also developed a recent approach based on the one described above due to the need for accurate folding of both natively ordered proteins and intrinsically disordered proteins calculations [108]. Their work demonstrated that the approach, which we describe above, was prone to produce proper ensembles for disordered peptides [29]. Their third approach is defined as

(90) $$G_{tr}\left(T\right)=\sum_{k=1}^{n}∆g_{tr,2}^{sc,k}\left(T\right)\alpha^{sc,k}+∆g_{tr,2}^{bb}\left(T\right)\sum_{k=1}^{n}\alpha^{bb,k}\;,\;\forall T$$

(91) $$G_{np}^{0}=0$$

This approach also failed in calculating proper results since it overestimated the helical propensity of disordered peptides. Therefore, they introduced a new approach, approach 4, where approach 1, which is explained in detail in Ref. [29], was applied for amino acids, such as Asparagine, Aspartic Acid, Isoleucine, Leucine, and Lysine, whereas approach 3 was applied for the backbone and the solvation of the remaining side chains. However, this approach partially corrects overestimation of helicity, thus showing no dramatic improvement over approach 3 [29].

### 2.9. The GBNSR6 Model

The GBNSR6 is a grid-based molecular surface implementation that uses the R6 variant of the Generalized Born implicit model. The R-6 (R6) integral allows the calculation of Born radii. GBNSR6 in AmberTools23 is integrated into MMPBSA.py and can be used to calculate the solvation free energy [109].

Izadi, Aguilar, and Onufriev used the GBNSR6 model to predict the protein–ligand binding energies. Specifically, the binding free energies of 15 small protein–ligand complexes obtained using GBNSR6 were examined by utilizing TIP3P and TIP4Ew OPC explicit solvent free energies as references. The root mean square deviation of GBNSR6 using TIP3P was found to be close to the error margin of explicit solvents. $\Delta \Delta G_{pol}$ calculated with GBNSR6 was closer to the calculations utilizing the OPC model. It was shown that almost all deviations were improved by simple uniform scaling of the set of Bondi radii. Large discrepancies were found between the binding and solvation energies using explicit models. Thus, the need for developing more accurate implicit solvent models was emphasized. We should note that it was stated that it would be more accurate to measure the model accuracy by comparing with experimental data [110].

In a study by Wang et al., the performance of various MM/PB(GB)SA approaches for predicting protein–protein interactions and protein–ligand binding structures was systematically evaluated using a dataset of 900 unique docking poses. The MM/PBSA method demonstrated superior pose ranking accuracy compared to MM/GBSA and Glide SP scoring, with the PB3 model achieving a success rate of 74%. Among the MM/GBSA approaches, the GB6 model was particularly notable, highlighting the effectiveness of the GBNSR6 implicit solvent model. Additionally, this work led to the development of the Fast Amber Rescoring for PPI Inhibitors (farPPI) web server, which enabled the efficient scoring of docking poses using MM/PB(GB)SA methods [111]. In the study by Forouzesh et al., the impact of different atomic radii parameterizations on binding free energy calculations was assessed using the Ras-Raf protein complex, and the resulting absolute binding free energies showed good agreement with experimental values. Subsequently, the performance of the GBNSR6 model was evaluated for predicting the binding free energy between the SARS-CoV-2 spike receptor binding domain (S RBD) and the human ACE2 receptor. When using the Bondi radii set, the calculated binding free energy was overestimated, while the OPT1 radii set led to an underestimation. The calculated ΔG_bind_ values for the SARS-CoV-2 S RBD–ACE2 complex deviated by approximately 4 kcal/mol from the experimental reference of approximately −10.6 kcal/mol. Errors in SPR (Surface Plasmon Resonance) modeling are inherent, resulting from instrumental, biochemical, and data analysis, and experiments are also prone to errors. Although this deviation is greater than the experimental uncertainty, it demonstrates that the MMGB/SA method provides qualitatively reliable results in this system due to the near-cancelation of the (ΔH) and entropy (−TΔS) terms [112].

Furthermore, Tolokh et al. studied the implicit water multipole GB (IWM-GB) model that was developed based on the GBNSR6. In addition to incorporating dipole water polarization, as in GBNSR6, this model also accounted for the multipolar field effects of water molecules within the first hydration shell of the solvent. The model showed results compatible with experimental data. Root mean squared error decreased by 12% compared to the usage of the TIP3P model. The IWM-GB model was examined in two sub-versions as IWM-GB WC and IWM-GB NC. While IWM-GB WC includes the polar–nonpolar interaction, IWM-GB NC does not. The charge hydration asymmetry (CHA) effect of the WC model was noted to be more suitable for achieving experimental results. Thus, the importance of the polar–nonpolar interactions in the model was demonstrated. The IWM-GB model does not take into account the hydrogen bridges formed by water molecules between two atoms in polypeptides [113].

## 3. Implicit Solvation Models in Molecular Quantum Mechanics

### 3.1. Classical Electrostatic Models

As mentioned above, the Born model was used to calculate the solvation energy of a spherical ion in a dielectric medium [114]. The model is based on classical electrostatics and remains valid at the point where the solvent is considered as a continuous dielectric medium [115]. The Born model offers a straightforward and intuitive formula for solvation energy, which is proportional to the square of the charge and inversely related to the ion’s radius [116]. However, its use is limited to spherical ions. It also does not take into account the shape of molecules or non-electrostatic factors such as cavitation. The Poisson–Boltzmann equation, considered an important model for electrostatic solvation, describes the electrostatic potential in a medium in which a charged solute is surrounded by a dielectric solution [114]. This equation can be expressed in its linear or nonlinear form, with the nonlinear form accounting for the Boltzmann distribution of mobile ions in the solvent. As a difference from the Born model, the PB equation incorporates both the fixed charges of the solute and the ionic strength of the surrounding medium, providing a more realistic description for electrolyte solutions. However, its numerical solution can be computationally demanding for large and complex biomolecular systems, motivating the development of faster approximations such as the GB model [117].

Generalized Born models, developed in the 1980s, build upon the Born model to accommodate molecules of various shapes [37]. This is accomplished by introducing effective Born radii, which estimate the solvation energy of a molecule by summing pairwise interactions between atomic charges [9]. The GB model is known to be computationally efficient and appropriate for large systems. As a result, it is quite a popular parameter model for molecular dynamics (MD) simulations [118]. The validity of this model is in doubt for some systems as it depends on the Born radii used and the pairwise interaction approximation [119].

### 3.2. Quantum Mechanical Continuum Models

The 1980s also saw an important step forward in implicit solvent model development. In a Polarizable Continuum Model (PCM), the solvent is treated as a polarizable continuum and the electrostatic solute–solvent interaction is estimated by solving the Poisson equation [120]. The PCM solvation energy is written as

(92) $$\Delta G_{solv}=\frac{1}{2}\int \rho \left(r\right)\phi_{reaction}\left(r\right)dr$$

Here, ρ(r) is the charge density of solute and $\phi reaction\left(r\right)$ is the reaction potential due to solvent. The Poisson equation is solved to obtain the reaction potential:

(93) $$\vec{\nabla }\cdot \left(\varepsilon \left(r\right)\vec{\nabla }\phi_{reaction}\left(r\right)\right)=-4\pi \rho \left(r\right)$$

where $\varepsilon \left(r\right)$ is the solvent dielectric constant. PCM gives a more accurate description of the solute–solvent interaction than the Born and GB models, since the detailed charge distribution of the solute is taken into account [12].

The Conductor-like Screening Model (COSMO) is a simplification of the PCM approach, which assumes that the solvent behaves as a conductor and has an infinite dielectric constant. This is based on an assumption that increases the computational efficiency of COSMO at the same time as preserving reasonable accuracy. Solvation energy in COSMO is described by [121]:

(94) $$\Delta G_{solv}=\frac{1}{2}\sum_{i}\sigma_{i}\phi_{i}$$

Here, $\sigma_{i}$ is the surface charge density at point i on the solute–solvent boundary, and $\phi_{i}$ is the electrostatic potential at point i; the surface charges are given by the solution of the matrix equation [122].

(95) Aσ=Bϕ

COSMO is commonly used method in DFT calculations due to its computational ease [123]. It has an infinite dielectric constant, however, and this is a perfect approximation for solvents in many cases but expands sources of error when using solvents with a lower dielectric constant (nonpolar ones) [124]. In contrast, for nonpolar systems, C L-based COSMO overestimates the solvation energy, which leads to less accurate predictions [125]. The underlying reason for this limitation is the inability of COSMO to describe the weak electrostatic and specific solute–solvent interactions (hydrogen bonding, van der Waals) that exist in nonpolar media. To circumvent these limitations, more comprehensive models such as COSMO-RS (Real Solvent) have been developed [13]. Extensions by statistical thermodynamics that take into account different solute/solvent interactions such as hydrogen bonding and van der Waals forces are built upon the original COSMO, which lead to the COSMO methods (continuum solvation COSMO) and the COSMO-RS [126]. Therefore, compared to QSPR, COSMO-RS has the potential to more accurately summarize predictions for a much broader range of solvents, such as ionic and nonpolar liquids. COSMO-RS is a versatile method and can also estimate thermodynamic properties such as activity coefficients, vapor–liquid equilibria, and partition coefficients [127].

Due to its simplicity and computational efficiency, COSMO is still commonly used for solvation studies in a high-dielectric and polar solvents, despite some discrepancies. Performance and scalability are important for high-throughput applications and DFT calculations where this is very useful. For higher accuracy, the use of more sophisticated models, such as COSMO-RS or SMD (Solvation Model based on Density), is recommended in systems with non-homogeneous, nonpolar solvents or complex solute–solvent interactions [128]. In fact, COSMO-RS uses statistical thermodynamics to account for some solute–solvent interactions, such as hydrogen bonding, van der Waals forces, and solvent–solvent interactions, whereas COSMO assumes the solvent acts as a conductor with an infinite dielectric constant [129]. For predicting thermodynamic parameters like activity coefficients, vapor–liquid equilibria, and solubility in a variety of solvents, including polar, nonpolar, and ionic liquids, COSMO-RS is therefore more accurate [130]. In order to account for non-electrostatic interactions, COSMO-RS incorporates a residual term into the electrostatic framework of COSMO. There are two primary components of the overall solvation energy in COSMO-RS [131]; when considering the electrostatic component side, this component is computed using the same methodology as in COSMO, which makes use of the electrostatic potentials $\sigma_{i}$ and surface charge densities $\phi_{i}$ [130]:

(96) $$∆G_{COSMO}=\frac{1}{2}\sum_{i}\sigma_{i}\phi_{i}$$

The solvent’s polarization effects are captured by this phrase. This component takes into consideration non-electrostatic interactions on the residual element side, including solvent–solvent interactions, van der Waals forces, and hydrogen bonding. The expression for the remaining energy is [130] as follows:

(97) $$∆G_{res}=\sum_{i,j}\Gamma_{i,j}\sigma_{i}\sigma_{j}$$

In this case, $\sigma_{i}$ and $\sigma_{j}$ are the surface charge densities at surface segments i and j, and $\Gamma_{ij}$ is the interaction energy between these segments [132]. The COSMO-RS model has a number of noteworthy benefits that make it an effective tool for solvation modeling and thermodynamic property prediction [129]. Its extensive application to a variety of solvents, including polar solvents like alcohols and water, nonpolar solvents like hexane and toluene, and even ionic liquids, is one of its main advantages [129].

In contrast to the original COSMO, which is mostly accurate for high-dielectric solvents and assumes an infinite dielectric constant, COSMO-RS takes into account particular solute–solvent interactions like solvent–solvent interactions, hydrogen bonding, and van der Waals forces [133]. This allows it to produce highly accurate predictions for systems like complex mixes or nonpolar solvents where these interactions are crucial [134]. The capacity of COSMO-RS to accurately forecast thermodynamic characteristics is another significant benefit [135]. It is extremely useful in drug discovery and (bio)chemical process design since it is frequently used to compute activity coefficients, vapor–liquid equilibria, solubility, and phase behavior [129]. COSMO-RS also achieves a balance between computing efficiency and accuracy [136]. The addition of residual factors for non-electrostatic interactions makes it more computationally demanding than COSMO, but it is still effective enough to manage large systems. For comparison purposes, traditional COSMO and COSMO-RS can be compared as follows [137].

The SMx models, which were developed from the 1990s to the 2000s, represent a family of semi-empirical solvation models that incorporate both electrostatic and non-electrostatic contributions [33]. These models are designed to be universal, allowing them to be applied across a variety of solvents and solutes [33]. The solvation energy in the SMx models is expressed as follows [33]:

(98) $$∆G_{solv}=∆G_{elec}+∆G_{non-elec}$$

or,

(99) $$G_{solv}=G_{elec}+G_{cav}+G_{disp}+G_{rep}$$

The electrostatic term $∆G_{elec}$ is calculated using a dielectric continuum model like PCM or GB, while the non-electrostatic term $∆G_{non-elec}$ encompasses cavitation, dispersion, and repulsion effects. The non-electrostatic term is formulated as follows [33]:

(100) $$∆G_{non-elec}=\gamma A+\alpha B+\beta \;$$

γ, α, and β are parameters, while A is the solvent accessible surface area. B is the cavity dispersion–solvent structure term (CDS), representing non-electrostatic dispersion and structural effects of the solvent on the solute. SMx models are frequently employed in quantum modeling and are renowned for their exceptional precision. However, the quality of the parameterization determines how accurate they are [33]. SM5.4, SM6, SM8, and SMD are among the models in the SMx family; they differ in their mathematical formulations and parameterizations [138]. To study certain solvation modeling issues, these variations are required [139]. The balance between efficiency and precision is an important factor [140]. Models such as SM5.4 and SM6 are particularly well-suited for the high-throughput screening of extensive molecular datasets, as they achieve computational efficiency through the use of simplified formulations for non-electrostatic contributions [141]. However, by incorporating more thorough parameterization and empirical terms—both of which are essential for precisely computing solvation free energy in complicated systems—models such as SM8 and SMD place an emphasis on accuracy [142]. Their broad compatibility with a wide range of solvents—including polar, nonpolar, protic, and aprotic media—and diverse solutes such as small organic molecules, ions, and biomacromolecules is another crucial factor, enabled by extensive parameterization and solvent-specific calibration [143].

Certain models are optimized for specific solutes, like ions or neutral molecules, or for specific solvent types, like water or organic solvents [33]. For instance, SM5.4 is especially made for tasks like analyzing neutral molecules in aqueous solution, whereas SMD is a general-purpose model that can handle a wide range of solvents and solutes, including ionic species [33]. Additionally, choosing a model is influenced by compatibility with quantum mechanical techniques [33]. As a matter of fact, the SMx models may be used to DFT and other quantum mechanical techniques, including semi-empirical techniques such as AM1 and PM3 [141]. Model selection is impacted by the theoretical level used in the computations (Table 1) [15]. For instance, SMD and DFT are commonly used for high-accuracy calculations, while SM5.4 and semi-empirical methods are often used for faster calculations [142]. Because of these variations in design and use, the SMx family is flexible and able to handle a variety of computational chemistry problems [15].

The Solvation Model based on Density (SMD) is a model that merges the advantages of PCM for electrostatics with empirical non-electrostatic terms [144]. SMD is intended for use with a broad spectrum of solvents and solutes, including both neutral molecules and ions. The solvation energy in SMD is represented as

(101) $$∆G_{solv}=∆G_{elec}+\gamma A+\alpha B+\beta$$

The electrostatic term $∆G_{elec}$ is derived from a dielectric continuum model, while the non-electrostatic terms γA + αB + β account for cavitation, dispersion, and repulsion effects. SMD is widely used to forecast solvation free energies and is compatible with a number of quantum mechanical techniques, such as DFT [145,146]. However, parameterization for various solvents affects its accuracy, just like it does for other SMx models [14]. The quality of the dataset used for parameterization determines the accuracy of the empirical parameters (γ, α and β), which are obtained from experimental data [33]. Because the settings have been well adjusted using a sizable database of experimental solvation free energies, SMD is extremely accurate for solvents like water and organic solvents [33]. However, parameterization might be less accurate for ionic liquids or less widely used solvents. SMD uses a parameterization technique that attempts to cover a wide range of solvents and solutes in order to overcome this restriction. With this method, a variety of experimental data, such as solvation free energies, activity coefficients, and partition coefficients, are fitted to empirical parameters [33].

It is important to recognize that the physicochemical characteristics of the solvent–solute system can substantially influence the accuracy of SMD predictions [33]. For example, certain solute–solvent interactions, such as hydrogen bonding or van der Waals forces, may be difficult for the model to describe in ionic liquids or strongly nonpolar solvents [147]. Apart from the difficulties in parameterization, the accuracy of SMD is also affected by the chosen quantum mechanical method [145]. Although DFT and semi-empirical approaches are compatible with SMD, the quality of the electrostatic and non-electrostatic terms may be impacted by the level [148]. High-level DFT simulations with precise electron density distributions, for example, typically produce better results than semi-empirical approaches, which might incorporate more approximations [148]. Despite these challenges, the versatility of the SMD model (ability to provide reliable solvation free energy estimates plays a critical role in drug discovery, particularly in predicting binding affinities, optimizing solubility and guiding lead compound selection), resulting from its broad applicability to diverse solvent environments and chemical systems, has made it one of the most widely used continuum solvation models [33].

### 3.3. Quantum-Centric Implicit Solvation

Merz’s team, working with IBM, demonstrated the first implicit-solvent quantum-chemistry simulations on real quantum devices by integrating the sample-based quantum diagonalization (SQD) framework with the integral equation formalism Polarizable Continuum Model (IEF-PCM) [149]. In their formulation, a quantum circuit prepares samples of a correlated wavefunction (via a Local Unitary Cluster Jastrow, LUCJ, ansatz); these samples define a reduced Hamiltonian that is then diagonalized classically, while the solvent reaction field is introduced through IEF-PCM as a self-consistent post-processing step. This hybrid workflow reaches solution-phase ground-state energies and solvation free energies without explicit water, closing a long-standing gap between gas-phase quantum demonstrations and realistic chemistry. The team executed SQD/cc-pVDZ IEF-PCM for water, methanol, ethanol, and methylamine on IBM quantum processors using active spaces that mapped to 27, 30, 41, and 52 qubits, respectively. Across these molecules, solvated energies tracked CASCI/IEF-PCM baselines as sampling increased, indicating that solvent-aware quantum runs can achieve chemically meaningful accuracy on current hardware. Technical ingredients included the use of self-consistent configuration recovery (S-CORE) to restore particle number and spin symmetries in noisy bitstrings and a truncated LUCJ ansatz parameterized from classical CC amplitudes, which are choices that make SQD comparatively robust on today’s devices. The solvent-aware SQD study extends a broader quantum-centric program from the same collaboration, which has progressed from gas-phase supramolecular interactions to embedding (DMET-SQD) for larger molecules. Together, these results map a credible scaling path. This involves using quantum sampling to capture strong correlation in a controllable active space, stitching in continuum solvation to reflect experimental conditions, and offloading the heavy linear algebra to classical solvers. Ongoing discussion in the literature contrasts SQD/QSCI against classical selected CI heuristics; the Merz–IBM work addresses practicality through sampling/batching strategies and noise recovery while demonstrating solution-phase targets on real QPUs.

## 4. Machine Learning-Augmented Implicit Solvent Models

Classical continuum approaches such as PB and GB, together with quantum–continuum frameworks like PCM, COSMO, and SMx, have long provided tractable routes to solvent energetics. Yet they still struggle with nonlocal many-body hydration effects, ion specificity, dielectric boundary ambiguity, and parameter transferability across chemistries and conformational states. Machine learning is now influencing implicit solvation along three complementary lines: physics-informed surrogates that learn to emulate continuum electrostatics; end-to-end models that learn an implicit potential of mean force directly from explicit solvent trajectories; and physics-guided hybrids that correct the remaining, systematic errors of fast PB/GB-type models. Each line targets a different bottleneck such as numerical cost, missing many-body solvent effects, or model bias so that they are best viewed as synergistic rather than competing approaches. In the first line, Poisson–Boltzmann-based machine learning (PBML) models are trained on thousands of biomolecular instances computed with high-order PB solvers, such as MIBPB, and then used to predict electrostatic solvation free energies for new structures with accuracy on par with, and often exceeding, traditional PB solvers at a fraction of the cost [150]. These PBML models provide a practical route to PB-level electrostatics for large-scale screening or long MD, where repeated PB calls would be prohibitive. Related physics-informed neural formulations are being explored to solve linear PBE directly, further improving the momentum toward ML-accelerated electrostatics. In the second line, end-to-end ML models learn an implicit solvent potential from explicit solvent data so that subsequent simulations can run without water. ISSNet, for example, trains a graph neural network to reproduce the solvent-averaged forces on peptide solutes and then drives MD with the learned PMF, recovering conformational thermodynamics more faithfully than GB/SA baselines [151]. A complementary study trained a DeepPot-SE model on alanine dipeptide in an average solvent environment; the learned model reproduced solute forces within ~0.4 kcal mol^−1^ Å^−1^ RMSD and the free-energy surface within <0.9 kcal mol^−1^ of explicit solvent MD [151]. Early evidence also suggests generalization beyond the training chemistries for short peptides using GNN-based schemes. The third line closes the loop with physics-guided hybrids that keep the continuum backbone but learn corrections where the physics is systematically approximate. A representative example is a physics-guided neural network (PGNN) built on GBNSR6 [152]. On the PDBbind v2016 benchmark, PGNN achieved a test RMSE of 4.08 kcal mol^−1^, improving compared to GraphConv (6.90) by about 40% and compared to AtomicConv (5.23) by about 20%. On the host–guest set, PGNN cut the GBNSR6 error from 8.35 to 2.05 kcal mol^−1^ (≈6.3 kcal mol^−1^ gain), illustrating how data-driven residual learning can deliver practical accuracy gains while retaining the speed and interpretability of GB.

A closely related hybrid idea is learning the residual between physics-based hydration free energies and the experiment as a lightweight post-processing step. Using graph-based featurization, such ML corrections reduced test-set RMSEs of fast GB predictions by about 50%, bringing them close to the accuracy of uncorrected TIP3P while adding negligible overhead. This is attractive when the goal is to maintain a standard PB/GB workflow but improve quantitative agreement [153,154] (and references therein). These ML trends complement physics-driven advances in implicit solvents. For example, the new Implicit Water Multipole GB (IWM-GB) framework introduces water multipoles into the GB formalism and reports clear accuracy gains relative to standard GB and competitive performance versus explicit water in relevant benchmarks, as explained above [113]. Such progress strengthens the physics baseline that ML methods can then refine further. Looking forward, the pragmatic best practice is likely to be hybridization, meaning the use of PBML-style surrogates to retain continuum interpretability and scalability; deploy end-to-end learned PMFs where explicit solvent thermodynamics must be captured at implicit-model cost; and apply residual ML corrections to close the remaining accuracy gap in the experiment. We anticipate the rapid maturation of uncertainty quantification, active-learning workflows that target “hard” chemistries for additional labeling, and consensus testing on community datasets, such as FreeSolv and host–guest suites, to standardize comparisons across methods.

## 5. Some Applications in Biology

**Figure 1:**
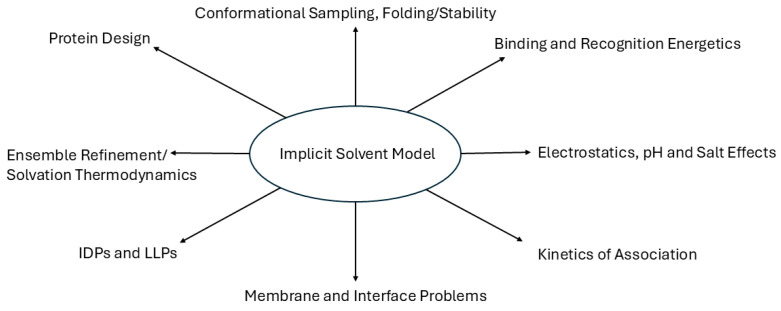
Some application areas of implicit solvent models in biophysics.

Implicit solvent models have been used in various subareas of biophysics (Figure 1). Intrinsically disordered proteins are proteins that do not have a stable three-dimensional structure [145]. Techniques such as X-ray crystallography and cryo-electron microscopy are insufficient in terms of explaining their disordered nature. Their properties are investigated with applications combined with implicit solvent models. Different dissolution times are observed by representing continuous products instead of individual atoms. When working with existing implicit solvent models, the formation of excessively collapsed disordered states in explicit solvent models has been observed because of comparisons made with FRET and SAXS experiments. The problem of overestimating the α-helix and β-sheet structures has been largely solved by the backbone torsional change based on the new generation NMR systems. For instance, the EEF1 model by Karplus and Lazaridis uses the solvation free energies in empirical runs adjusted according to the degree of buriedness of the protein functional groups [86]. It calculates the electrostatic interactions in solution with the distance-dependent dielectric constant by neutralizing the ionic side chains. The EEF1 model separates the free energy of a protein in solution into two main components: intramolecular energy and solvation free energy. The interaction of an atom with a solvent is measured by how much the surrounding atoms restrict solvent access [80]. The ABSINTH model designed by Vitalis and Pappu models these proteins better; EEF1 and ABSINTH differ in the choice of solvation groups and the way they measure solvent accessibility. This model has a two-component approach to describe the transfer of solute to the continuum, the modeling of DMFI (direct mean-field interaction), and the screening of polar interactions. The polar and nonpolar parts of the transfer process are handled using reference solvation free energies for the solvation groups [28].

Validation studies of force fields are being carried out in implicit solvent frameworks. In the study by Mandacı et al., α-synuclein was simulated using REMD along with an implicit water model (AMBER ff99SB/OBC implicit solvent model) and compared to classical MD techniques in explicit water with various force fields such as a99SB*-ILDN/TIP3P, a99SB-ILDN/TIP4P-D, a03ws, a99SB, CHARMM C22*, CHARMM36m, and a99SB-disp. The α-helix, 3_10_-helix, β-sheet, and turn structure probabilities obtained were compared with the radius of gyration values to evaluate the effects of force field and technique differences on epitope site identification [154]. Pietrek et al. used the Amber99SB*-ILDN-q force field and the TIP3P/TIP4P-D water models to fragment α-synuclein into overlapping fragments of five amino acids each. After sampling local structures with REMD, full-length structures were generated using a hierarchical fragment-assembly algorithm and validated using NMR chemical shifts and SAXS data [155]. The OPLS4 force field, developed by Lu et al., was reparametrized compared to OPLS3e to increase hydration accuracy, reduce the overstabilization of salt bridges, and improve sulfur interactions. This force field was extensively validated with small molecule hydration and solvent transfer free energies, acetate–guanidine ion pair interaction energies, and Asp/Glu pKa predictions [156].

The accurate calculation of protein–ligand binding energy is an essential step in drug design. There are many implicit solvent models used in this field. Some of them are PCM, S-GB (Surface Generalized Born), COSMO, GBNSR6 (Generalized Born R6 version), and the PB model [157]. In a study conducted by Katkova et al., the hydration, solvation, and desolvation energies calculated with these models were compared with the results calculated using explicit solvent models and experimental data. They focused on polar components rather than nonpolar components. As a result, although Poisson–Boltzmann and GBNSR6 achieved the most accurate results in terms of accuracy in calculating desolvation energies, GBNSR6 model stood out in terms of speed. However, it can be seen that better parameter selections are still needed in desolvation energy calculations. This is because the choice of atomic radii and the charges have a strong influence on the calculation of desolvation energy. None of these models could provide chemical accuracy (error level less than 1 kcal/mol) [157].

Guo, Zuojun et al. used Coloumb area approximation (CFA) and level-set variational implicit-solvent model (VISM) to determine protein binding surfaces. Most small molecules tend to bind to hydrophobic pockets with complex topology on the surface of proteins. In this method, the dissolution process of molecules was described by minimizing the solvation free energy of solute–solvent interfaces. Surface tension, electrostatic, and van der Waals interactions were calculated using an implicit solvent model. In this way, the depth, volume, and hydrophobic properties of binding pockets could be rapidly determined [158]. Furthermore, Feig and co-workers performed DNA and DNA–protein simulations using the GBMV model. The effect of salt presence was also evaluated. The obtained results were analyzed by comparing them with explicit solvent simulations and experimental data. Simulations performed with the default radius set in CHARMM were calculated using root mean square deviations. The DNA structure remained stable throughout the simulation. The simulated structure was quite similar to the experimental structure. When the standard helical parameters were examined, it was seen that they are in good agreement with the average helical parameters obtained from explicit solvent simulations, but the standard deviations were two times larger. Since implicit solvent models captured the effects of water molecules in less detail, a higher degree of oscillation was observed [159].

In a study by Prabhu et al., a hybrid implicit water solvation model was developed for DNA and RNA molecular simulations. Specifically, the finite-difference Poisson–Boltzmann (FDPB) and GB models were generally applied in cases where molecules with small net charges and ionic strength effects were not dominant. This hybrid model was developed due to the lack of detailed examination of the cases where molecules carried high charges. In the FDBP model, the ionic strength was set to zero, thus reducing it to the finite-difference Poisson (PDB) model. Ions were explicitly treated as solvents. Since ions constitute a smaller part of the total number of the system, this explicit approach did not increase the computational power significantly. It was observed that long-term stability was provided in simulations performed on B-DNA dodecamers, decamers, and tetraloop RNA molecules. Similar results were obtained for explicit water simulations. In the analyses performed with the CURVES DNA structure analysis program, it was observed that there was no statistically significant difference in 24 helicoidal and base parameters. The implicit solvent model accurately simulated the transition from A to B, and similar final stable B structures were achieved in previous studies. Results were obtained faster compared to the usage of explicit solvent models [160].

## 6. Future Perspectives

Implicit solvent models have been useful in biomolecular simulations and quantum mechanical calculations, yet ongoing advances in methodologies and our understanding of solvation phenomena promise to drive the field forward. A key future direction involves the integration of artificial intelligence to refine parameterization, improve transferability across diverse environments, and accelerate the development of next-generation hybrid models. Physics-guided neural networks and data-driven approaches can further enhance the predictive accuracy and computational efficiency of implicit solvation methods. Quantum computing and multi-scale modeling frameworks offer exciting opportunities for simulating large, complex biomolecular systems with accuracy. Combining quantum models with high-level electronic structure methods will enable the study of challenging processes such as enzymatic catalysis and the behavior of intrinsically disordered proteins in solution. Improved representations of solvent heterogeneity, the explicit treatment of ion effects, and dynamic solvent–solute interfaces are likely to overcome limitations in modeling solvation and entropic effects.

## 7. Conclusions

Implicit solvent models remain indispensable for connecting molecular-scale physics to mesoscopic biological function at a tractable cost. Across this review, we surveyed classical continuum electrostatics (PB/GB), nonpolar and cavity/dispersion formulations, widely used numerical implementations (DelPhi, APBS), quantum–continuum approaches (PCM, COSMO, SMx/SMD), and specialized frameworks for challenging regimes such as intrinsically disordered proteins (e.g., ABSINTH and transfer free energy methods). We also discussed practical applications in protein–ligand binding, nucleic acids, and disordered ensembles, where implicit models enable rapid hypothesis testing, large design sweeps, and long-time sampling, which would be prohibitive with an explicit solvent. The core strength of implicit models is combining efficiency with interpretability: they expose clear physical knobs—including dielectric contrast, radii, surface definitions, salt screening—that map cleanly onto mechanistic hypotheses and can be stress-tested. At the same time, limitations persist. Specific solvent structure (bridging waters, ion–pairing), heterogeneous environments, and entropic contributions are only approximately captured, and quantitative accuracy is sensitive to parameters and boundary conventions. These trade-offs argue for fit-for-purpose use rather than one-size-fits-all prescriptions. For high-throughput ranking and pose filtering, modern GB/PB implementations, such as GBNSR6 and PBSA, offer robust signals when parameters are reported transparently and sensitivity analyses are performed. For IDPs and other flexible systems, IDP-aware Hamiltonians and transfer free energy treatments better preserve ensemble properties and experimental observables. For electronic structure, PCM/COSMO/SMD enable solution-phase reactivity and spectroscopy with balanced cost, provided non-electrostatic terms and cavity definitions are validated for the solvent and chemotypes at hand. Two frontiers are reshaping the landscape. First, ML increasingly augments continuum baselines: PB surrogates deliver PB-level accuracy at GB-like cost; learned implicit potentials of mean force capture solvent-averaged forces for MD; and physics-guided residual models reduce systematic errors in binding energetics while preserving interpretability. Second, quantum-centric workflows that couple continuum solvation, such as IEF-PCM, to quantum sampling on real hardware demonstrate a viable path toward realistic solution-phase quantum chemistry. We expect hybridization to dominate the field via continuum cores refined by improved physics, such as multipolar water responses, ML correctors with uncertainty quantification and active learning, and quantum–continuum modules for chemically demanding steps. As these pieces mature, implicit solvent modeling will continue to provide the speed, interpretability, and scope required to interrogate complex biophysical problems, while steadily narrowing the accuracy gap with experiments through principled, data-aware improvements.

## Figures and Tables

**Table 1:** A summary of SMx models and their characteristics.

Model	Key Feature	Mathematical Approach	Advantages	Applications	Mathematical Approach
SM 5.4	Designed for fast and efficient calculations.	Uses the GB model for electrostatic terms. Non-electrostatic terms (cavitation, dispersion, repulsion) are modeled using surface area and volume.	Computationally efficient. Suitable for high-throughput screening.	Studying neutral molecules in aqueous solutions.	Electrostatics: GB. Non-electrostatics: ASA/volume-based (cavitation, dispersion, repulsion).
SM 6	An improved version of SM5.4. Includes more accurate parameterization.	Uses PCM or GB for electrostatic terms. Non-electrostatic terms are modeled with more detailed parameters.	Higher accuracy than SM5.4. Still computationally efficient.	Studying ionic solutions and solubility of organic molecules.	Electrostatics: PCM/GB. Non-electrostatics: Parametrized (detailed parameters).
SM 7	Focuses on improving non-electrostatic terms.	Uses PCM for electrostatic terms. Non-electrostatic terms are modeled with advanced empirical parameters.	Better accuracy for non-electrostatic effects.	Solvation of polar and nonpolar molecules in various solvents.	Electrostatics: PCM Non-electrostatics: Improved parametrization for cavitation, dispersion, repulsion
SM 8	A universal model optimized for both ionic and neutral molecules.	Uses PCM for electrostatic terms. Non-electrostatic terms are modeled using surface area, volume and advanced empirical parameters.	High accuracy for a wide range of solvents and solutes.	Drug design, ionic solutions, and solubility of organic molecules.	Electrostatics: PCM. Non-electrostatics: ASA/volume + advanced empirical parameters.
SMD	The most advanced SMx model. Designed as a universal solvation model.	Uses PCM for electrostatic terms. Non-electrostatic terms are modeled using surface area, volume and empirical parameters (γγ, αα, ββ).	High accuracy across a wide range of solvents and solutes. Compatible with DFT.	Drug design, materials science, environmental chemistry (e.g., solubility of pollutants).	Electrostatics: PCM. Non-electrostatics: ASA/volume + empirical parameters (γγ, αα, ββ).
SM 12	An extension of SMD with improved parameterization.	Uses PCM for electrostatic terms. Non-electrostatic terms are modeled with more refined empirical parameters.	Enhanced accuracy for specific solvent-solute systems.	High-precision calculations for solvation free energies in complex systems.	Electrostatics: PCM. Non-electrostatics: ASA/volume + refined parameters.
SM x-NP	Designed for nonpolar solvents and solutes.	Uses GB or PCM for electrostatic terms. Non-electrostatic terms are modeled with parameters optimized for nonpolar interactions.	Accurate for nonpolar systems.	Studying organic semiconductors and polymers in nonpolar solvents.	Electrostatics: GB/PCM. Non-electrostatics: Parameters optimized for nonpolar interactions.
SM x-IL	Tailored for ionic liquids.	Uses PCM for electrostatic terms. Non-electrostatic terms are modeled with parameters optimized for ionic liquids.	High accuracy for ionic liquid systems.	Studying solvation and reactivity in ionic liquids	Electrostatics: PCM. Non-electrostatics: Parameters optimized for ionic liquids.

## Data Availability

No new data were created or analyzed in this study. Data sharing is not applicable to this article.

## Abbreviations

The following abbreviations are used in this manuscript:

ABSINTH	self-assembly of biomolecules studied by an implicit, novel, and tunable Hamiltonian
APBS	Adaptive Poisson–Boltzmann Solver
ASA	Accessible surface area
CFA	Coulomb area approximation
CHA	The charge hydration asymmetry
COSMO	Conductor-like Screening Model
COSMO-RS	Conductor-like Screening Model-Real Solvent
DMFI	Direct mean-field interaction
FDBB	Finite-Difference Poisson–Boltzmann
GB	Generalized Born model
GBNSR6	Generalized Born R6 version
IDPs	Intrinsically disordered proteins
IWM-GB	Implicit water multipole Generalized Born
MD	Molecular dynamics
ML	Machine learning
PB	Poisson–Boltzmann equation
PCM	Polarizable Continuum Model
PDB	Finite-difference Poisson
PGNN	Physics-Guided Neural Network
SAV	Solvent-accessible volume
S-GB	Surface Generalized Born
SMD	Solvation Model based on Density
VISM	Level-Set Variational Implicit-Solvent Model
BE	Boundary Element
MBE	Multigrid Boundary Element
VHS	Exposed Volume of the Hydration Shell
UNRES	United Residue Force Field
SQD	Sample-Based Quantum Diagonalization
IEF-PCM	Integral Equation Formalism Polarizable Continuum Model
PBML	Poisson–Boltzmann–based machine learning
